# Allergic and Mixed Rhinitis: Diagnosis and Natural Evolution

**DOI:** 10.3390/jcm8112019

**Published:** 2019-11-19

**Authors:** Justin C. Greiwe, Jonathan A. Bernstein

**Affiliations:** 1 Bernstein Allergy Group 8444 Winton Road, Cincinnati, OH 45231, USA; jcgreiwe@gmail.com; 2 Department of Internal Medicine, Division of Immunology/Allergy Section, The University of Cincinnati College of Medicine, 231 Albert Sabin Way, ML#563, Cincinnati, OH 45267-0563, USA

**Keywords:** allergic rhinitis, mixed rhinitis, diagnosis, allergy testing, guidelines, quality of life, medication responsiveness, rhinitis subtypes

## Abstract

Chronic rhinitis (CR) is divided into two main categories: allergic rhinitis (AR) and nonallergic rhinitis (NAR). These conditions are more recognizable to an experienced clinician, as they can be more clearly demarcated diagnostically. However, an additional 30% to 50% of patients with CR might have an overlap of NAR and AR, referred to as mixed rhinitis (MR). Progress in elucidating the pathophysiologic mechanisms behind MR and NAR has been made in the past several years, and there are now several guidelines published to assist the clinician in accurately diagnosing AR, NAR, and MR. Clinical history and subjective symptoms can provide clues for differentiating AR from MR and NAR, but allergy testing is recommended to confirm these conditions. Progress in accurately diagnosing patients with CR will be made as studies incorporate subjective (i.e., validated questionnaires such as the irritant index questionnaire (IIQ), medication responsiveness, and quality-of-life tools) and objective (i.e., nasal cytologic testing, nasal provocation, and biomarkers) methods characterizing rhinitis subtypes.

## 1. Introduction

Chronic rhinitis (CR) is an often-trivialized condition that is frequently poorly characterized, resulting in an incorrect diagnosis and subsequent treatment recommendations. As a result of its improper classification, a spectrum of comorbidities including sinusitis, otitis, conjunctivitis, eustachian tube disorders, chronic cough, obstructive sleep apnea, headaches, and fatigue can occur, leading to a poor quality of life, increased healthcare utilization, and an unnecessary economic burden to society [1]. Symptoms include, but are not limited to, nasal congestion, sinus pressure, itching (usually involving the nose, mouth, eyes, or throat), puffy/swollen eyelids, post-nasal drainage, rhinorrhea, sneezing, and cough. Establishing the correct diagnosis is the first step toward better medical management. Classically, CR is divided into two main categories: allergic rhinitis (AR) and nonallergic rhinitis (NAR) [2]. These conditions are more recognizable to an experienced clinician as they can be more clearly demarcated diagnostically. However, an additional 30% to 50% of patients with CR might have an overlap of NAR and AR referred to as MR (mixed rhinitis) that can be more challenging to diagnose [3]. This condition is often referred to as “the more difficult form or allergic rhinitis to treat”. Therefore, an accurate diagnosis of allergic and mixed rhinitis (MR) requires a better understanding of the various rhinitis phenotypes and endotypes. A detailed differential diagnosis of CR is provided in Table 1; however, this chapter will focus on the allergic and MR phenotypes [1].

### 1.1. Allergic Rhinitis

Allergic rhinitis is a ubiquitous condition estimated to affect approximately 20% of the U.S. population and can be triggered by seasonal allergens, perennial allergens, or both [4]. Seasonal AR symptoms can occur in spring, summer, and early fall, and are caused by allergic sensitivity to airborne pollens from grass, trees, and weeds or to mold spores. Perennial AR sufferers experience symptoms year-round triggered by dust mites, pet hair or dander, cockroaches, or mold. The pathogenesis of AR involves several effector cell types, cytokines, and bioactive mediators that contribute to the inflammatory process, leading to a biphasic inflammatory reaction characterized clinically as immediate (early) and late-phase symptoms. Patients genetically predisposed to developing allergic rhinitis develop specific immunoglobulin E (sIgE) to an inciting allergen (e.g., grass pollen) that binds to high-affinity IgE receptors on mast cells and basophils. The early phase clinical response occurs after these previously sensitized mast cells are activated by exposure to the specific relevant allergen, resulting in the release of pre-formed and newly formed bioactive mediators. These mediators bind to receptors on blood vessels, resulting in physiologic changes that manifest as nasal congestion; anterior rhinorrhea; or posterior nasal drainage, sneezing, and itching. The late-phase response typically begins 4–8 h after allergen exposure and involves cellular migration and activation of lymphocytes, eosinophils, and basophils that release of cytokines and chemotactic factors, resulting in additional pro-inflammatory changes that manifest as persistent nasal obstruction, anterior and posterior rhinorrhea, fatigue, and malaise [5].

### 1.2. Non-Allergic Rhinitis 

Whereas confirmation of AR requires positive skin-prick or serologic sIgE testing that correlates with symptoms after exposure to the sensitizing aeroallergen, a diagnosis of NAR is made after negative skin-prick or serum sIgE testing to aeroallergens. Patients with NAR often exhibit similar symptoms to AR patients but complain less of sneezing and itching and more of worsening nasal congestion, rhinorrhea, sinus headaches, and post-nasal drip [6,7]. Triggers for NAR patients include irritants (cigarette smoke, perfumes, cleaning products), weather changes (barometric pressure, temperature), and other strong odors. However, many NAR patients are unable to identify specific triggers, which may reflect exposure opportunity or lack of awareness of what is causing their symptoms. Although NAR consists of a broad category of rhinitis subtypes, vasomotor rhinitis is the most common form and constitutes the majority of cases [1]. Vasomotor rhinitis (VMR) and NAR are commonly used interchangeably because a consensus definition has yet to be agreed upon due to a poor understanding of the underlying mechanism(s) of action and lack of objective diagnostic biomarkers for these conditions [8]. However, several studies now strongly suggest that the pathophysiologic mechanism of VMR involves neurogenic pathways leading to a hypercholinergic response [9,10]. Stimulation of transient response potential (TRP) calcium ion channels in the nose by nonspecific environmental factors such as temperature, mechanical, osmotic or chemicals and irritants is known to activate nasal afferent nerve fibers that lead to their depolarization and release of neuropeptides (substance P, neurokinin A, calcitonin gene related peptide), as well as signaling through the central nervous leading to subsequent efferent responses (i.e., glandular, and/or vascular responses manifested as rhinorrhea and nasal congestion, respectively) [11,12]. Additional nasal symptoms that can be caused by the local release of the neuropeptides include itching, cough and sinus pain. [12,13].

### 1.3. Mixed Rhinitis 

There is another subset of patients who present with CR symptoms that worsen in response to both allergic and non-allergic triggers referred to as MR. This third category of CR is fairly common, with prevalence rates ranging anywhere from 35–50% [14]. A retrospective questionnaire analysis conducted by the National Rhinitis Classification Task Force (NRCTF) reported the prevalence of chronic rhinitis patients in allergy practices with “pure” allergic rhinitis is 43%, “pure” nonallergic rhinitis is 23%, and MR is 34% [3,15]. A subsequent study, which involved 3398 patients with CR, found that >90% of patients with seasonal AR reported symptoms in response to at least one nonallergic trigger. Temperature changes (71.4%), tobacco smoke (60.8%), perfumes (56.4%), and cleaning products (37.9%) were the most frequently reported symptoms, suggesting MR is a common clinical presentation [16]. Although MR shares similar characteristics to AR, neurogenic mechanistic pathways are also involved, which explain their increased sensitivities to nonspecific stimuli and decreased response to medications such as second-generation antihistamines and nasal corticosteroids. Regardless of the terminology used to describe these patients, their clinical presentation suggests that the pathophysiologic features of this condition is much different than AR [17]. 

### 1.4. Diagnosis

Distinguishing between the various rhinitis phenotypes can be confusing as the clinical presentation for each can be similar. Understanding the subtle differences between these conditions allows for a more accurate diagnostic approach, leading to better symptom control and improvement of quality of life. Bernstein et al. reported that when an objective irritant index questionnaire is used to diagnose chronic rhinitis patients, almost 25% of patients previously assigned a physician diagnosis of AR were reclassified as MR because of their high total irritant burden [14]. Reclassification of AR to MR revealed that patients with MR had more frequent and severe symptoms, as well as an increase in physician-diagnosed asthma. Thus, although clinical symptoms such as congestion, rhinorrhea, sneezing, or post-nasal drainage can overlap between AR and MR phenotypes, other characteristics such as triggers, response to treatment, and prevalence of comorbidities such as sinusitis and asthma are often significantly different. 

A comprehensive past medical history and focused physical exam serves as the cornerstone for a complete workup that can provide clues for what rhinitis phenotype the patient may have. The focus of the exam should be on the nasal mucosa but also include the oropharynx, eyes, ears, neck, lungs, and skin. Classic physical findings suggestive of seasonal AR include “allergic shiners” (i.e., discoloration under the lower eyelids related to vasodilation or venous congestion); “allergic salute” or horizontal nasal crease across the lower half of the bridge of the nose caused by repeated upward rubbing of the tip of the nose by the palm of the hand; mouth breathing; conjunctivitis; and edematous, pale, boggy nasal mucosa. In addition, AR is often associated with other comorbid allergic conditions such as asthma, atopic dermatitis, and food allergy. Physical exam findings for perennial AR and NAR can be more subtle with normal or erythematous nasal mucosa, often with evidence of prominent post-nasal drip manifesting as “cobblestoning”, which refers to streaks of lymphoid tissue on the posterior pharynx. Fiber-optic nasal endoscopy provides the best visualization of intranasal structures, but this more invasive exam is not always available or necessary. Many patients with chronic rhinitis, especially MR and NAR, have a decreased sense of smell and sometimes distorted taste. A persistent absence of smell after proper treatment could suggest nasal polyps, which are often visualized by nasal exam if they are advanced but can easily be missed if they are not anterior and prolapsed. Finally, patients with MR and NAR also experience ear popping and pressure due to eustachian tube dysfunction, manifesting as retraction of the tympanic membranes that are caused by increased negative pressure in the middle ear. 

There seems to be distinguishing characteristics that can clue the observer on which rhinitis phenotype the patient might be suffering from. A number of studies have attempted to classify clinically reproducible features of each rhinitis phenotype to aid in differentiating between AR and NAR. Di Lorenzo et al. evaluated 1511 adult patients with chronic rhinitis using various diagnostic parameters and determined that AR patients have more severe symptoms with increased sneezing, nasal pruritus, and recurrent conjunctivitis [18]. Compared to their NAR counterparts, AR patients also had higher nasal eosinophil counts, higher peak nasal inspiratory flow measurements, higher blood eosinophil counts, and a more impressive visual analog scale of nasal symptoms [18]. Allergic rhinitis patients often have a well-defined family history of allergies and, if they endorse symptoms in response to cats and dogs, are typically found to be sensitized to these animals by skin or specific IgE serologic testing. In contrast, NAR patients were older and predominately female with more nasal obstruction and posterior rhinorrhea, along with more frequent episodes of recurring headaches, as well as olfactory dysfunction [18,19]. A synopsis of these characteristics is summarized in Table 2. However, relying solely on clinical characteristics for an accurate diagnosis can be confusing due to overlapping characteristics of rhinitis subtypes and therefore, as discussed below, testing to determine sensitization to aeroallergens is necessary for an accurate diagnosis of AR, MR, or NAR [20].

### 1.5. Triggers

Establishing the diagnosis of AR requires symptoms upon exposure to sensitizing aeroallergen, whereas NAR seems to be a diagnosis of exclusion based on the absence of allergic triggers and negative confirmatory skin or serologic testing to aeroallergens. With limited diagnostic testing specific to NAR, focusing on pertinent odorant and irritant triggers has been considered an important diagnostic criterion for establishing the correct diagnosis. Some have even postulated that patients with NAR have abnormalities in their olfactory transduction pathway, leading to pathologic changes in the olfactory receptors or mucosa, making them more susceptible to these triggers [21,22]. Rezvani et al. attempted to further elucidate the role of olfaction in CR subtypes using olfactory threshold measurements in a subset of CR patients but found no differences between patients with AR, NAR, or MR [23]. Contrary to many NAR patients’ belief that they have a hyper-acute sense of smell, this data suggests neither impaired nor overactive olfaction is a feature of NAR. Common NAR triggers include changes in the environment (dry cold air, humidity, barometric pressure), airborne irritants including odors and fumes (cologne, perfume, cleaning products, cigarette smoke, diesel, and car exhaust), certain medications (beta-blockers, aspirin, and other non-steroidal anti-inflammatory drugs), dietary factors (spicy food, alcohol), sexual arousal, exercise, strong emotions or stress, and hormone levels [24].

An environmental exposure chamber (EEC) models has been used to simulate weather conditions in a controlled setting to objectively diagnose NAR subjects and ultimately to investigate novel NAR therapies [25]. Others have used cold dry air provocation as a tool to differentiate NAR patients from healthy control subjects [26]. The results of these studies suggest that the EEC model and nasal provocation to non-allergic triggers provides a consistent and reliable method to phenotype NAR patients and could be used to investigate disease mechanisms and novel therapies for NAR in the future [25,26]. Although establishing pertinent irritant triggers is an important aspect for the evaluation of CR subtypes, this diagnostic criterion has its limitations. A recent study found that a significant proportion of NAR patients (64%) had a low non-allergic trigger burden, suggesting that a large subgroup of NAR patients may either not recognize irritant triggers as causing or aggravating their rhinitis symptoms or that symptoms in response to non-allergic triggers should not be considered an essential part of the diagnostic workup for all NAR patients [14].

## 2. Questionnaires 

In an attempt to better evaluate clinical control of CR, the Rhinitis Control Assessment Test was developed, which is outlined in Table 3 [27]. This simple, standardized, validated patient-completed questionnaire is a useful tool to help physicians identify patients with uncontrolled CR and facilitate treatment and subjective progress over time [28]. The higher the Rhinitis Control Assessment Test score, the better the patient’s control (scores range from 6 to 30, and any score of ≤21 indicates poor control) [27]. The minimally important difference was found to be 3 points, and a change of at least 3 points is considered clinically meaningful [27].

Although the clinical history and physical exam can be helpful aspects of the diagnostic workup, several studies suggest that that clinical symptoms and triggers alone might not be as reliable for differentiating between rhinitis subtypes. Incorporating patient-centric questionnaires into the patient evaluation is one way clinicians have attempted to more reliably characterize CR subtypes. Brandt and Bernstein distributed a questionnaire listing symptoms and common allergic and nonallergic triggers to 100 randomly selected patients with CR symptoms that ultimately was highly predictive of NAR on the basis of responses concerning triggers, but less helpful in the diagnosis of AR [19]. Patients whose symptoms began at the age of 45 years, without a parental history of allergies, with the occurrence of symptoms in the presence of perfumes and fragrances, but none in the presence of cats and cat hair or while being outdoors during the spring, had a 98% probability of having NAR prior to any type of testing to assess atopy [27]. Subsequent questionnaires developed by Bernstein et al. attempted to further classify CR using an irritant index questionnaire (IIQ) consisting of a 21-item survey that rated the degree to which nonallergic triggers induced rhinitis symptoms (0–10-point scale) [14]. A total of 656 patients were enrolled in the study, of which 404 had a physician diagnosis of AR (61.5%), 129 had MR (19.7%), and 123 had NAR (18.8%). Each participant’s original physician diagnosis of AR, MR, or NAR was subject to reclassification on the basis of a quantitative IIQ score and could be reclassified into four separate groups:

1. Patients with a physician diagnosis of AR or MR and a low IIQ score were reclassified as having low-burden AR.
2. Patients with a physician diagnosis of AR or MR and a high IIQ score were reclassified as having high-burden AR.
3. Patients with a physician diagnosis of NAR and a low IIQ score were reclassified as having low-burden NAR.
4. Patients with a physician diagnosis of NAR and a high IIQ score were reclassified as having high- burden NAR.

Results revealed that 52% of all patients with a physician diagnosis of AR and MR fit into the high-burden AR group. Of note, 47% of those with a physician diagnosis of AR were later reclassified as having high-burden AR. These findings suggest that patients with AR experience a high burden of non-allergic triggers that are not being recognized by the patient or treating clinician. The high irritant burden rhinitis group seemed to have more severe and frequent rhinitis symptoms and a significantly greater prevalence of physician diagnosed asthma, suggesting that the IIQ successfully reclassified rhinitis patients into distinct subgroups with unique clinical differences that may represent more severe phenotypes than previously recognized by current diagnostic modalities [14,24]. This patient-centric questionnaire represents a useful tool to help identify patients with a high irritant trigger burden, which is especially important because there are no standardized lists of nonallergic triggers currently used to establish a diagnosis of MR.

### 2.1. Skin and Serum Testing

As demonstrated in previous studies highlighted above, depending exclusively on the medical history to determine the appropriate rhinitis subtype can be confusing. Patients often refer to their diagnosis as “hay fever” or AR on the basis of pre-conceived notions of CR and triggers emphasized in the news and by advertisements. Because of the ambiguity of clinical histories, objective assessment by allergen skin prick testing (SPT) or specific in vitro IgE testing is essential. Skin testing involves introducing measured amounts of seasonal and perennial aeroallergens, indigenous to the geographic location the patient resides, in conjunction with a positive histamine and negative saline control onto the skin followed by a prick with a special bifurcated needle. If the SPT is negative but the clinical history is concerning for aeroallergen sensitivity, intracutaneous testing (ICT) can also be performed. However, several studies have found that ICT has a high sensitivity but is less specific than SPT, and as a result might lead to false positive testing if previous percutaneous testing was unremarkable [29,30]. The goal of SPT is to elicit an immediate wheal and flare response within 15 min after application, confirming the patient has specific IgE antibody directed to the allergen on high affinity (FcER1) IgE receptors located on cutaneous mast cells. Cross-linking of the allergen to the specific IgE antibody binding regions results in mast cell activation and release of bioactive mediators, resulting in the physiologic characteristics of AR [24]. 

Serum-specific IgE antibody testing to aeroallergens is often ordered when the patient is dermatographic, has a severe skin disease (e.g., poorly controlled atopic dermatitis, chronic urticaria), or is on medications that interfere with the histamine response (e.g., antihistamines, tricyclic antidepressants) necessary for skin test interpretation. It is important to remember that a positive skin or serum sIgE test only implies sensitization and should always be correlated with the patient’s reaction history upon exposure to that allergen before the diagnosis of “allergy” can be made. 

Although the testing modalities currently available are typically accurate, discrepancies can occur, as in the case of patients with localized AR (LAR) or “entopic” rhinitis. This subset of patients has negative skin and/or serologic testing to aeroallergens but has all the classic symptoms of AR upon exposure to a specific allergic trigger that can be reproduced with nasal provocation to the specific allergen. Furthermore, they respond to traditional treatments used for AR such as nasal corticosteroids and second generation antihistamines. Rondón et al. estimates that local AR affects 25.7% of the rhinitis population and more than 47% of patients previously diagnosed with NAR, but these numbers have not been consistently confirmed by other investigators [24,31]. Given the unique nature of this disease process, the question arises as to whether LAR is a specific condition or just the initial presentation for the natural course of AR [32].

A wide number of methods for classifying and diagnosing rhinitis subtypes have been proposed, including nasal cytology, peak nasal inspiratory flow rates, anterior rhinomanometry, acoustic rhinometry, irritant index scales, specific biomarkers, nasal provocation testing with environmental exposure chambers, and nasolaryngoscopy [32]. All of these proposed testing methods are an attempt by investigators to more accurately subtype CR in order to develop more focused treatment plans for the patient and improve their clinical outcomes. Thus far, many of these tools are not readily available to clinicians and have also been hampered by varying degrees of reliability and cost. Tests with no proven diagnostic validity include cytotoxic tests, specific IgG testing, provocation-neutralization, electrodermal testing, applied kinesiology, iridology, and hair analysis [33].

### 2.2. Imaging

Imaging is not typically part of the initial diagnostic workup of CR; however, in refractory or more severe cases, computerized tomography (CT) scan of the sinuses can help further elucidate sinus anatomy and/or structural changes, including obstruction or narrowing of the osteomeatal complexes and septal deviation, as well as identify the presence of nasal polyps. Sinus plain films are not recommended as they are less sensitive than sinus CT scans and are not reliable enough to assist with clinical decision making. 

### 2.3. Comorbidities 

It is important to recognize that CR can be associated with various comorbidities, which can significantly impact management and further impair quality of life. Commonly associated comorbidities include acute and chronic sinusitis, tension and migraine headaches, asthma, chronic cough, conjunctivitis, Eustachian tube dysfunction, otitis media with or without effusion, nasal polyps, hearing impairment, changes in smell and taste, nasal dyspnea, obstructive sleep apnea, and other sleep disturbances/related complications resulting in fatigue [24]. Complications stemming from poorly controlled CR include poor cognitive functioning, sleep loss/daytime fatigue, reduced school/workplace productivity, and absenteeism. Chronic cough secondary to post nasal drainage is a frequently an unrecognized complication of CR that often gets confused with asthma, bronchitis, or gastroesophageal reflux [34,35,36,37]. Interestingly, Singh et al. found that comorbid CR was significantly associated with an increased risk of 30 day asthma- and chronic obstructive pulmonary disease-related hospital readmission [38]. These findings highlight the important relationship between the upper and lower respiratory tracts and how the appropriate recognition and treatment of the correct CR subtype can lead to reduced early hospital readmissions and subsequent health care costs [38].

## 3. Conclusions

Progress in elucidating the pathophysiologic mechanisms of MR and NAR has been made in the past several years, and there are now several guidelines published to assist clinicians in accurately diagnosing rhinitis subtypes [33,39]. Clinical history and subjective symptoms can provide clues for differentiating AR from MR and NAR, but allergy testing is recommended to confirm these conditions. Progress in accurately diagnosing patients with CR will be made as studies incorporate subjective (i.e., validated questionnaires such as the IIQ, medication responsiveness, and quality-of-life tools) and objective (i.e., nasal cytologic testing, nasal provocation, and biomarkers) methods characterizing rhinitis subtypes [14]. The studies included in this article demonstrate the clinical utility of standardized, validated questionnaires, as well as the use of EEC models to objectively characterize CR subtypes, leading to a more accurate diagnosis and focused treatment. Although these modalities are the first steps in providing better insight into the underlying mechanisms of CR, further investigation is required to develop more accurate and clinically accessible tools.

## Figures and Tables

**Table 1:** Differential diagnosis of chronic rhinitis *, #.

Seasonal allergic rhinitis
Perennial allergic rhinitis
Non-allergic rhinitis with eosinophil syndrome (NARES)
Vasomotor rhinitis (idiopathic or irritant induced rhinitis) with and without triggers
Local allergic rhinitis (also known as entopic rhinitis)
Drug-induced rhinitis
(Topical nasal decongestants, oral contraceptives, nonsteroidal anti-inflammatory drugs, antihypertensive medications, β-blockers)
Infectious rhinitis
Occupational or work-related rhinitis

* Excluding structural blockages or chronic sinusitis; # adapted from [1].

**Table 2:** Distinguishing features of nonallergic rhinitis and allergic rhinitis #.

Nonallergic Rhinitis	Allergic Rhinitis
- Onset of symptoms later in life, more common after age 20 - No indication of a familial pattern - More common in females - Perennial symptoms in nature with very little seasonal variation * - Negative aeroallergen skin testing and/or serum immunoglobulin E (IgE) testing - Broad range of irritant triggers - Symptoms include: Nasal congestion Postnasal drainage with or without cough Infrequent eye complaints Minimal itching - Physical exam (more variable) Nasal mucosa can be normal with increased clear watery secretions, may be erythematous or atrophic	- Usually presents in childhood - Persuasive family history of atopy (asthma, rhinitis, and atopic dermatitis) - Affects females and males equally - Most have seasonal exacerbation of symptoms - Positive aeroallergen skin testing and/or serum IgE testing - Aeroallergen triggers - Symptoms include: Congestion, sneezing, rhinorrhea, and nasal itch Ocular conjunctivitis, watering, and itch - Physical exam Nasal mucosa edematous, pale, and boggy Allergic shiners (i.e., dark areas under the eyes)

* For nonallergic rhinitis (NAR), patients often attribute seasonal worsening of symptoms on aeroallergen triggers (i.e., tree and ragweed). These seasonal variations are not pollen-related, but rather due to changes in weather (i.e., temperature, humidity, barometric pressure) causing acute worsening of perennial symptoms. *#* Adapted from [16].

**Table 3:** Rhinitis Control Assessment Test (RCAT) questionnaire * #.

1. During the past week, how often did you have nasal congestion?
Never (5) Rarely (4) Sometimes (3) Often (2) Extremely Often (1)
2. During the past week, how often did you sneeze?
Never (5) Rarely (4) Sometimes (3) Often (2) Extremely Often (1)
3. During the past week, how often did you have watery eyes?
Never (5) Rarely (4) Sometimes (3) Often (2) Extremely Often (1)
4. During the past week, to what extent did your nasal or other allergy symptoms interfere with your sleep?
Never (5) Rarely (4) Sometimes (3) Often (2) Extremely Often (1)
5. During the past week, how often did you avoid any activities (for example, visiting a house with a dog or cat, gardening) because of your nasal or other allergy symptoms?
Never (5) Rarely (4) Sometimes (3) Often (2) Extremely Often (1)
6. During the past week, how well were your nasal or other allergy symptoms controlled?
Never (5) Rarely (4) Sometimes (3) Often (2) Extremely Often (1)

* Based on the five-point Likert scale (“Never” to “Extremely Often”) with a 1 week recall period for each item; Rhinitis Control Assessment Test scores range from 6 to 30, with any score of ≤21 indicating poor control. # Adapted from [23].
